# Hydration Mechanism of Solid Waste Gelling Materials Containing Semi-Dry Desulfurization Ash

**DOI:** 10.3390/gels11030193

**Published:** 2025-03-11

**Authors:** Yunyun Li, Siqi Zhang, Meixiang Huang, Guodong Yang, Jiajie Li, Mengqi Ma, Wentao Hu, Wen Ni

**Affiliations:** Key Laboratory of Resource-Oriented Treatment of Industrial Pollutants, School of Resource and Safety Engineering, University of Science and Technology Beijing, Beijing 100083, China; lyyustb@163.com (Y.L.); 13657814664@163.com (M.H.); 17866710223@163.com (G.Y.); jiajieli@ustb.edu.cn (J.L.); mq10706@163.com (M.M.); wthu010@ustb.edu.cn (W.H.); niwen@ces.ustb.edu.cn (W.N.)

**Keywords:** semi-dry desulfurization ash, blast furnace slag, mixed grinding, solid waste gelling materials

## Abstract

This study investigated the feasibility of using semi-dry desulfurization ash (DA) in combination with blast furnace slag (BFS) to prepare gelling materials, aiming to improve the resource utilization of DA. The effects of DA dosage and mechanical grinding on the compressive strength and hydration mechanism of BFS-DA gelling materials were investigated. The results showed that the optimum BFS-DA ratio was 60:40, and the compressive strengths were 14.21 MPa, 20.24 MPa, 43.50 MPa, and 46.27 MPa at 3, 7, 28, and 56 days, respectively. Mechanical grinding greatly improved the activity of the gel materials, with the greatest increase in compressive strength at 3, 7, 28, and 90 days for the BFS and DA mixed milled for 30 min, with increases of 89.86%, 66.36%, 24.56%, and 25.68%, respectively, and compressive strength of 26.22 MPa, 35.6 MPa, 58.33 MPa, and 63.97 MPa, respectively. The cumulative heat of hydration of BFS-DA slurry was about 120 J/g. The hydration mechanism showed that the main hydration products formed were ettringite, C-S-H gel, AFm, and Friedel’s salt. Calcium sulfite in DA was participated in the hydration, and a new hydration product, Ca_4_Al_2_O_6_SO_3_·11H_2_O, was formed. DA can be effectively used to prepare BFS-based gelling materials, and its performance meets the requirements of GB/T 28294-2024 standard, which provides a potential solution for the utilization of DA resources and the reduction in the impact on the environment.

## 1. Introduction

The emission of flue gases from the combustion of fossil fuels has been a global concern [1,2]. Coal-fired power generation, industrial boilers and sintering plants consume large amounts of fossil fuels every year and are the key industries for SO_2_ emissions [3,4,5]. Reducing the SO_2_ environmental pollution problem is an ongoing concern worldwide. At present, flue gas desulfurization processes mainly include the wet, dry, and semi-dry desulfurization of flue gas. Among them, comparing the different technologies, in wet desulfurization, equipment corrosion reduced by 50–70%, water consumption reduced by 80%, the investment cost reduced by about 30%, with fewer dry waste products, simple processing, less capital investment, and other significant advantages, and it has become the main method to mitigate the environmental problems caused by flue gas emissions [6,7,8]. However, semi-dry flue gas desulphurization technology is similar to other traditional flue gas desulphurization processes in that it produces de ash (DA) by-products. According to the database, the annual production of semi-dry flue gas desulphurization ash in China is about 20 million tons [9]. The production of DA reaches 13,076 tons in 2023, and the cumulative volume will reach billions of tons, making it the third-largest solid waste after fly ash and desulfurization gypsum. The composition of DA from steel mills is complex and variable, including CaSO_3_, CaSO_4_, CaCO_3_, f-CaO, CaCl_2_, and oxides such as Si, Mg, Fe, and Al [10]. Therefore, if DA is not managed properly, it leads to the transfer of SO_2_ to the air and soil, causing serious secondary environmental pollution of land and groundwater resources. With the complex composition of DA, it has low activity when used as a construction raw material, and components such as CaSO_3_, f-CaO, and CaCl_2_ lead to the degradation of the performance of construction materials. Nowadays, the research mainly focuses on the basic properties such as the physicochemical properties of dry desulfurization ash [11]. Studies have reported that DA was combined with steel slag and ordinary Portland cement (OPC) for the preparation of gelling materials, but it still needs to be mixed with admixtures to improve the early hydration process, and additionally, less than 20% of DA was mixed [12]. DA in the preparation of cementitious materials is still less reported.

Blast furnace slag (BFS) is a by-product of the blast furnace ironmaking process. Gelling materials containing blast furnace slag have made an important contribution to the sustainable green development of the cement industry. Cement production accounts for approximately 8% of global CO_2_ emissions [13]. BFS is used as a supplementary gelling material to produce slag cement, which is an environmentally friendly water-hard gelling material that not only reduces the proportion of clinker, but is also widely used in large-scale construction projects. Blast furnace slag itself has low activity, and the main active excitation methods at present are chemical activation, mechanical force activation, thermal activation, microwave activation, and so on [14]. The increase in the specific surface area of blast furnace slag after mechanical milling, the amorphization of mineral particles, strain enhancement, preferential dissolution of selected crystalline surfaces, structural disorder, and changes in microscopic morphology have led to a great improvement in the activity of blast furnace slag [15,16]. In addition, the synergistic preparation of gelling materials from BFS with other solid wastes has been widely reported. The utilization of blast furnace slag with steel slag and desulfurization gypsum can be used to fill gelling materials. The strength of blast furnace slag-based fillings is 2–3 times higher than that of cemented fillings. The gelling materials prepared from blast furnace slag and phosphogypsum for road base materials have good mechanical properties and durability, and the compressive strength reaches 41 MPa [17]. Guo et al. [14] studied a composite cementitious material of granulated blast furnace slag, steel slag, and calcium carbide slag, with a compressive strength of 60 MPa at 28 days. The key role of BFS in gelling material systems is to provide reactive silicon (aluminum) oxide tetrahedra that depolymerize and re-polymerize to form silicoaluminate gels, a process that typically requires alkali activation and sulfate activation. This process usually requires alkali activation and sulfate activation, where alkali activation accelerates the depolymerization of the glassy phase in the BFS, and sulfate activation is the formation of sulfate-phase minerals by combining the sulfate with Al, Si, and Ca in the system, as well as C-(A)-S-H. The two activation effects promote each other and the hydration continues until a dynamic equilibrium is reached. From the analysis of the chemical components of DA, it can provide an alkaline environment and sulfate for the hydration of cementitious materials [18].

**Figure 1:**
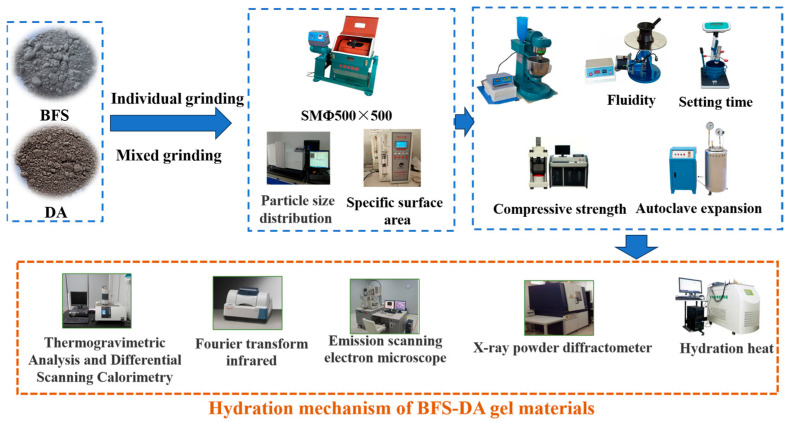
Experimental process.

In this study, BFS and DA were used to synergize the preparation of all-solid-waste gelling materials, and the ratio of BFS to DA was investigated in terms of compressive strength and fluidity of the slurry, as well as the enhancement of the activity of the gelling materials by different mechanical milling methods was examined. In addition, the hydration mechanism of the BFS-DA gel material was also investigated by thermal hydration test, X-ray diffraction (XRD), Fourier transform infrared spectroscopy (FTIR), scanning electron microscopy (SEM-EDX), and thermogravimetric analysis-differential scanning calorimetry (TG-DSC). The research roadmap is shown in Figure 1. This work will promote the resource utilization of desulfurization ash and accelerate its practical application in engineering construction.

## 2. Results and Discussion

### 2.1. Results of Gelling Materials with Different DA Dosages

**Figure 2:**
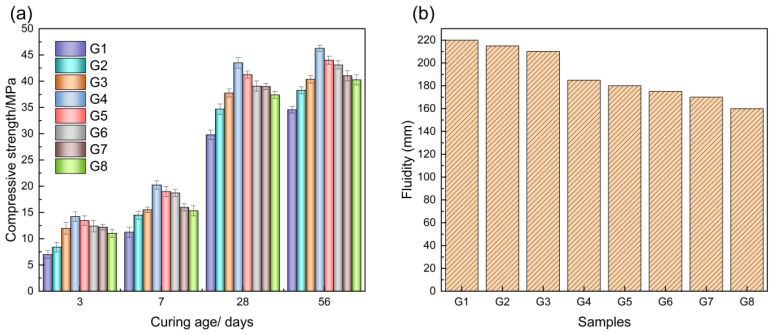
Compressive strength and fluidity results of gelling materials with different dosages of DA: (**a**) compressive strength; (**b**) fluidity.

The results of a compressive strength analysis of paste specimens of gelling materials with different dosages of desulfurization ash at different curing ages are shown in Figure 2. The compressive strength of the specimens showed a tendency of increasing and then decreasing with the increase in the dosage of DA, which contains a large amount of calcium hydroxide, which can play the role of alkali excitation for the slag. In addition, the sulfate ions in DA can form sulfate phase hydration products to provide strength for the system. When the dosage of DA is lower than 30%, the excitation effect of DA on BFS is not significant. When the DA dosage was 40% and the BFS dosage was 60%, the compressive strength at each curing age was the highest, and the compressive strengths at 3 d, 7 d, 28 d, and 56 d were 14.21 MPa, 20.24 MPa, 43.50 MPa, and 46.27 MPa, respectively. The compressive strengths were gradually decreased when the DA dosage was greater than 50%, in which the reduction in the compressive strengths at the late stage of curing was greater than that at the early stage of curing. This is due to the reduction in BFS dosage, the active silica–alumina–oxygen tetrahedron is reduced, and cannot form a sufficient amount of hydration products to provide strength support for the later stage.

As can be seen from Figure 2b, the fluidity of the gelling material paste decreases gradually with the increase in the DA dosage, and the fluidity of all groups reaches more than 160 mm. The distribution of fine particles of DA is finer compared with that of BFS, with D10 and D50 of DA being 1.65 μm and 11.2 μm, respectively, and those of BFS being 4.03 μm and 27.4 μm, respectively. The fluidity of the paste is affected by the fineness of raw materials and the particle grading. When DA dosage increases, the finer particles increase the specific surface area of gelling materials, so the fluidity gradually decreases. At 25–45% of DA dosage, the flow of gel material paste can be satisfied with most of the common concrete construction.

### 2.2. Mechanical Grinding to Enhance the Activity of Gelling Materials

#### 2.2.1. Specific Surface Area of DA at Different Grinding Times (Individual Grinding of DA)

**Figure 3:**
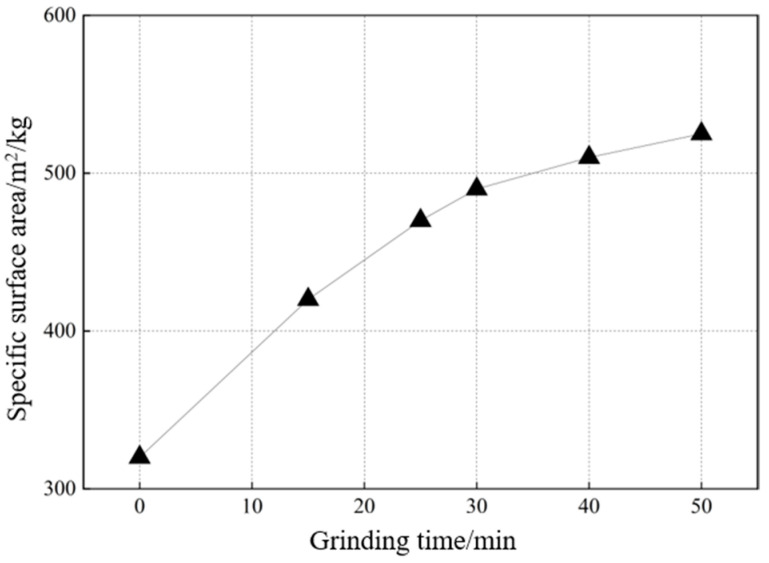
Specific surface area of DA with different grinding times.

The activity of the cementitious material was enhanced by mechanical grinding. The results of the specific surface area of DA ground alone are shown in Figure 3. It can be seen that DA has better grindability, and the specific surface can reach 420 m^2^/kg at 15 min and 490 m^2^/kg at 30 min. However, it only grows to 510 m^2^/kg when the grinding time is increased to 40 min. It is worth noting that the specific surface area of DA does not increase linearly with the extension of the grinding time. This is because when DA is ground too finely, the surface energy is too large and the interparticle forces increase, which in turn leads to agglomeration. As the surface area increases, the agglomeration between particles becomes more pronounced and the rate of increase in specific surface area slows down. Continuing to increase the grinding time leads to an increase in grinding energy consumption and production costs, which directly affects the economic benefits. Therefore, DA grinding for 30 min is appropriate.

#### 2.2.2. Particle Size Distribution of BFS-DA with Different Grinding Times (Mixed Grinding)

**Figure 4:**
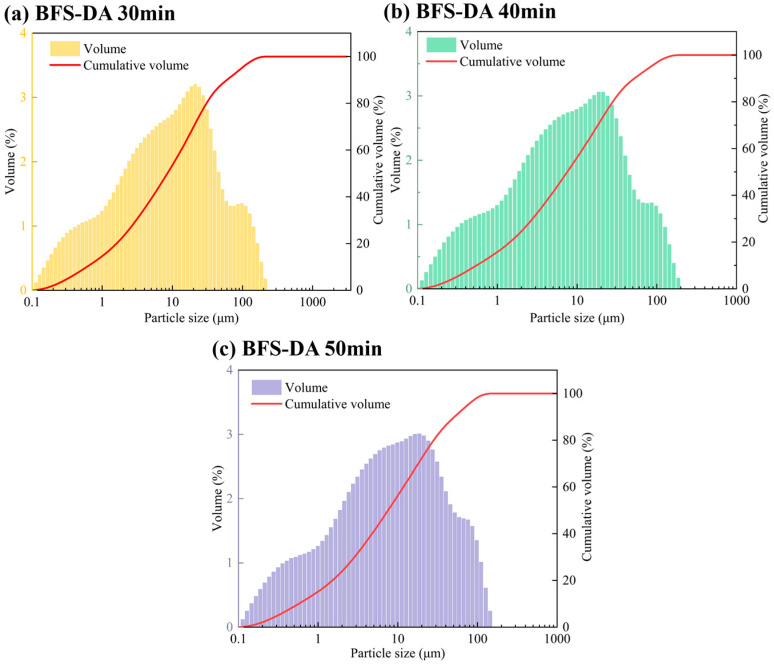
Particle size distribution of BFS-DA gelling materials with different grinding times.

Table 1 and Figure 4 show the effect of different mixed grinding time on the specific surface area and particle size of the BFS-DA system gelling materials. The specific surface area of the BFS-DA system gelling materials shows a trend of increasing and then decreasing with the increase in the grinding time, which is the same with the results of grinding DA individually. The specific surface area of BFS-DA system cementitious materials reached 580 m^2^/kg at 30 min of mixed grinding time; when the grinding time was extended to 40 min, the specific surface area increased to 591 m^2^/kg; and when the grinding time was extended to 50 min, the specific surface area decreased to 578 m^2^/kg. The D10, D50, and D90 of the BFS-DA system gelling materials with 40 min of mixed grinding were smaller than those of the BFS-DA system gelling materials with 30 min of mixed grinding and 50 min of mixed grinding. This phenomenon shows that although the content of fine particles increases gradually with the extension of the grinding time, the fine particles are prone to agglomeration under the extrusion of the grinding media, which leads to the reduction in specific surface area [19].

#### 2.2.3. Compressive Strength

**Figure 5:**
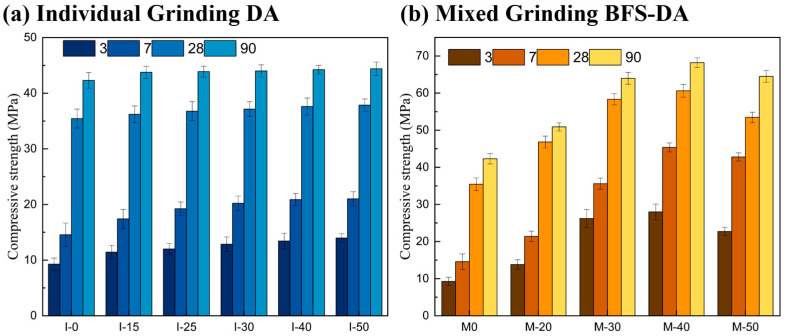
The effect of different grinding processes on the compressive strength of pastes.

The effect of different grinding processes on the compressive strength of BFS-DA gelling materials is shown in Figure 5. From Figure 5a, it can be seen that grinding DA individually is favorable to increase the compressive strength at early stage, while it has little effect on the compressive strength at the end of 28 days. DA has been mechanically milled and the increase in fines has resulted in a slight improvement in the early mechanical properties of the material. Mixed grinding can significantly improve the mechanical properties of BFS-DA gelling materials compared to grinding DA individually. The increase in compressive strength was 48.98%, 46.88%, 32.10%, and 20.30% for 3, 7, 28, and 90 d, respectively, after 20 min of mixed grinding. Under mixed grinding for 30 min, the maximum increase in compressive strength was 89.86%, 66.36%, 24.56%, and 25.68% for 3, 7, 28, and 90 d. The compressive strength reached 26.22 MPa, 35.6 MPa, 58.33 MPa, and 63.97 MPa, respectively. Under mixed grinding for 40 min, the increase in strength at the later stages of M-40 was significantly decreasing, and the 28 d and 90 d compressive strengths increased by 3.91% and 6.61% compared with M-30 specimens. Mixed grinding results in a larger contact area between the particles and uniform dispersion, which enhances the volcanic ash activity of BFS-DA, and thus the strength of the hydration process [20]. The compressive strength at all ages gradually decreased at 50 min of mixed grinding. This might be due to the long grinding time, which led to the distortion of the crystal structure of the raw material particles and the destruction of the active components [21,22]. In addition, it is known from Section 2.2.2 that the agglomeration phenomenon makes the specific surface area of BFS-DA gelling materials decrease at 50 min of mixed grinding, which then affects the contact area with water and reduces the reaction rate, and as a result, the strength of performance decreases [23].

#### 2.2.4. Setting Times and Stability

Table 2 shows that the mixed grinding BFS-DA gelling materials obviously shorten the initial setting time, and the measured setting time of the BFS-DA gelling materials slurry meets the indexes of composite gelling materials in the Chinese national standard GB/T 28294-2024 [24]; i.e., the initial setting time should not be less than 45 min, and the final setting time should not be more than 600 min. In addition, according to the test results of autoclave expansion and soundness, all of them meet the requirements of the standards and specifications.

#### 2.2.5. Hydration Heat with Different Grinding Times

**Figure 6:**
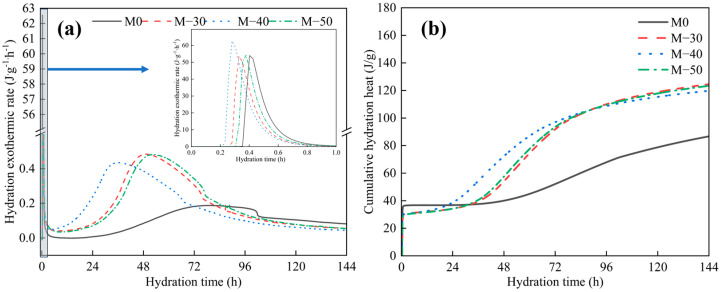
The hydration heat of BFS-DA gelling materials: (**a**) normalized heat flow; (**b**) cumulative hydration heat.

Figure 6 shows the hydration exothermic rate and cumulative heat of hydration of BFS-DA pastes with different mixing and milling times. With reference to the hydration of cement, the hydration of BFS-DA pastes is divided into five stages: initial, induction, acceleration, deceleration, and steady state [25]. As can be seen from Figure 6a, mixed grinding can significantly shorten the appearance time of the first heat release peak. DA contains a large amount of Ca(OH)_2_, and at room temperature, the pH of a saturated Ca(OH)_2_ solution is about 12.5. The alkaline environment accelerates the dissolution of the silicate-aluminate network of BFS. Increasing the time of mixed grinding promoted the uniform distribution of BFS and DA microparticles, which accelerated the hydration of the BFS-DA pastes. Mixed grinding for 40 min increased the first heat release peak, and the first heat release peak increased by 10.55 J/(g·h) compared with that of M0. In addition, it also shows a long acceleration period with a second wide low heat release peak at 18–72 h. This is consistent with the observation in the study of alkaline sulfate activated slag ternary system [26,27]. The maximum growth rate of cumulative hydration heat of BFS-DA pastes grows at the maximum rate for mixed grinding 40 min (Figure 6b). Prolonging the mixed grinding time, the agglomeration of microparticles was detrimental to the dissolution of the silico-aluminate network, and the formation of an impermeable silico-aluminate thin layer on the surface of BFS inhibited the further hydration of BFS. As a result, the compressive strength at all ages was reduced. The cumulative hydration heat of BFS-DA pastes was about 123.1 J/g, which was 59.9% lower compared with that of OPC (the cumulative heat of hydration was about 310 J/g) [28]. This indicates that BFS-DA gelling materials can be used to prepare concrete with relatively low heat of hydration, which can reduce the risk of infrastructure mass concrete due to thermal expansion.

### 2.3. Hydration Process

#### 2.3.1. XRD

**Figure 7:**
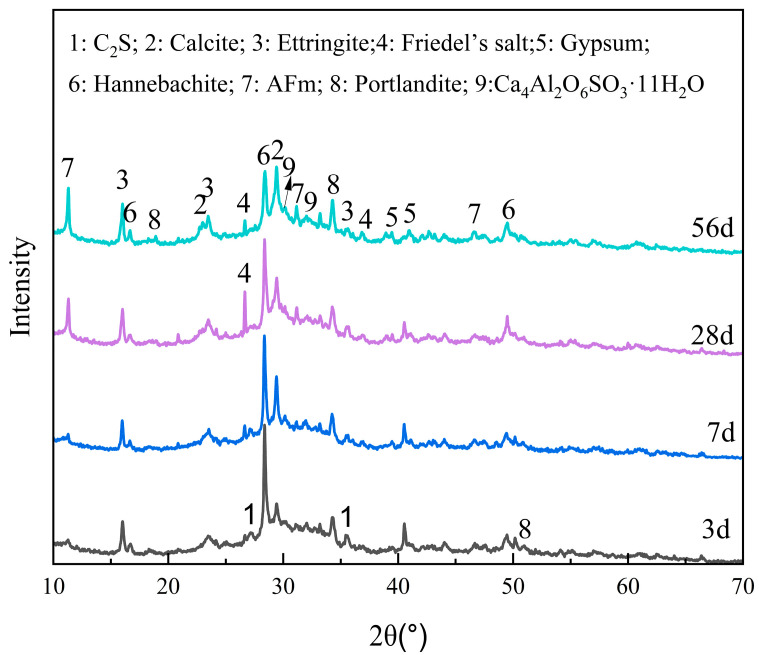
The XRD pattern of pastes at different curing age.

The XRD patterns of the BFS-DA pastes at different curing ages are shown in Figure 7. BFS-DA pastes contain material phases such as ettringite, AFm, Calcite, Friedel’s salt, gypsum, portlandite, and hannebachite. Ettringite, a common hydration product of gelling materials [29], appeared as an ettringite diffraction peak at 3 days of hydration, and AFm began to appear at 7 d hydration, and all of their peak intensities increased during the curing period. In addition, diffraction peaks of Friedel’s salt were observed at 28 d hydration, which was due to the small amount of Cl in the DA. The decrease in the peaks of Hannebachite (CaSO_3_∙0.5H_2_O) and gypsum originated from DA suggests that these minerals are involved in the hydration reaction. Hannebachite hydration with aluminate can produce Ca_4_Al_2_O_6_SO_3_·11H_2_O, similarly to the product of AFm. The diffraction peaks of calcite are gradually enhanced with widening as the curing age increases. This is also the location of the characteristic diffraction peaks of C-S-H gel. The silica–oxygen tetrahedral depolymerization in the BFS forms C-S-H gel with Ca^2+^ and OH^−^ in the paste, which is the main reason for the enhancement and broadening of the peak at 2θ of 28–30° [30].

#### 2.3.2. SEM-EDX

**Figure 8:**
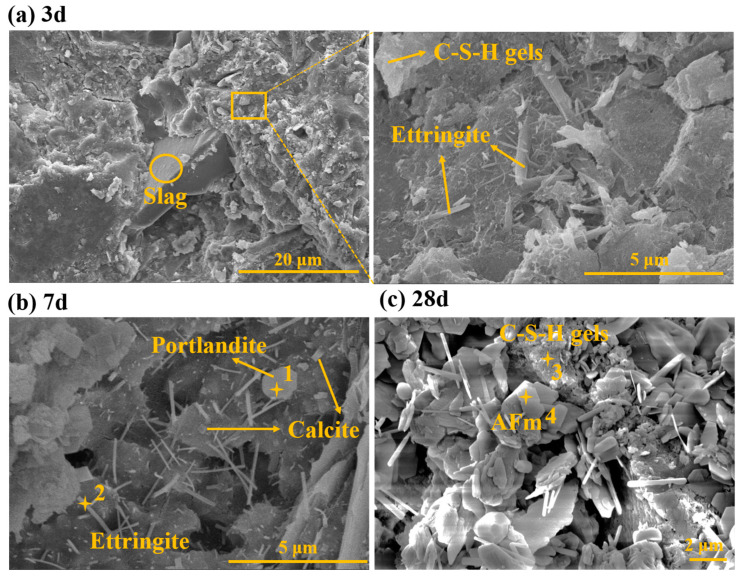
SEM images of BFS-DA gelling materials at curing age of 3 d, 7 d, and 28 d.

Figure 8 shows micrographs of BFS-DA gelling materials hydrated for 3, 7, and 28 days. Energy dispersive X-ray spectroscopy (EDX) was performed to analyze the elemental composition of the hydrated phase, and the results are shown in Table 3. There were unreacted raw materials in the paste after curing 3 d. Needle-like ettringite and amorphous C-S-H gel were also observed (Figure 8a). The development of the AFt crystals is still incomplete and the production of hydration products is relatively low, resulting in an overall looser structure. This is the main reason for the low early compressive strength [31]. After curing 7 d in Figure 8b, it can be seen that the ettringite in the slurry has needles changing to long columns and closely bonded together with the C-S-H gel. In addition, hexagonal platelets of portlandite and rhombohedral calcite are also observed. Figure 8c after curing for 28 d, the hydration products become significantly more abundant, and a large number of irregular flakes of AFm are observed in addition to the ettringite and C-S-H gel. It is worth noting that the morphology of the AFm differs slightly from that of previous reports, in which it was reported that Ca_4_Al_2_O_6_SO_3_·11H_2_O has similar morphology to AFm, so it is possible that Ca_4_Al_2_O_6_SO_3_·11H_2_O and AFm are mixed. All of these crystal form interstices were filled with C-S-H gel, and no portlandite was observed, probably covered by the main hydration products.

#### 2.3.3. FTIR

**Figure 9:**
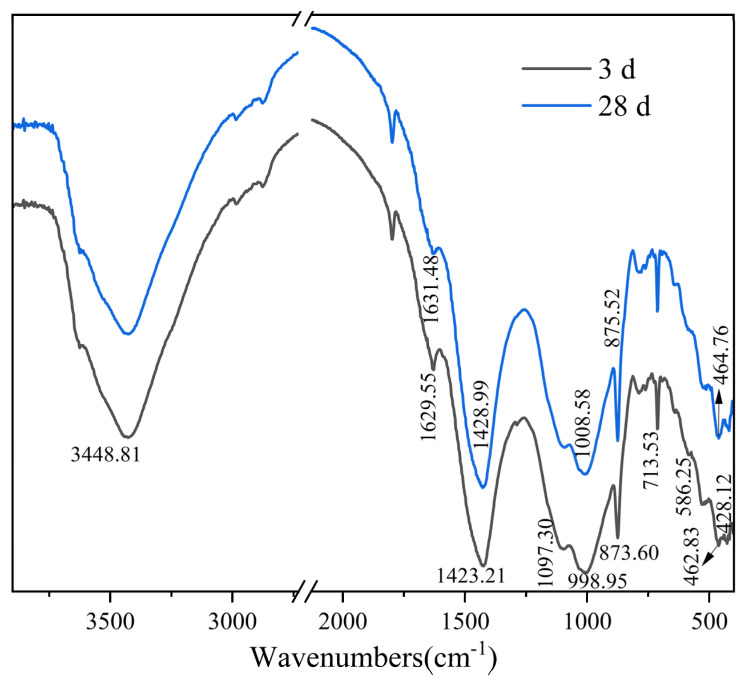
FTIR spectra of pastes at 3 d and 28 d.

The FTIR spectrum of the paste of DA-BFS gelling materials net is shown in Figure 9. 3448.81 cm^−1^ absorption peaks can be attributed to the O-H bond deformation vibrations, and the near 1629 cm^−1^ are related to the O-H bond stretching vibrations. These are related to hydration products, namely, C-S-H gel, AFt, AFm, and Friedel’s salt. The asymmetric stretching vibration and bending vibration of CO_3_^2−^ at 1423.21 cm^−1^ and 873.6 cm^−1^ are attributed to calcite in DA. It is also proved in XRD results for calcite. Peaks at 1097.3 cm^−1^ and 873.60 cm^−1^ are related to the stretching vibration of the Si-O-Si bond attributed to C-S-H gel. The peak at 586.52 cm^−1^ is related to the stretching vibration of the Si-O-Al bond, which is due to the substitution of Al for Si in the BFS, and the gradual breaking of the Si-O-Al bond as the hydration reaction proceeds [32]. The symmetric telescopic vibration associated with SO_3_^2−^ at 998.95 cm^−1^ is shifted to higher wave numbers, which suggests a change in the presence of SO_3_^2−^ and the formation of Ca_4_Al_2_O_6_SO_3_·11H_2_O [33]. The absorption peaks at 713.53 cm^−1^ are related to the bending vibration of SO_4_^2−^ attributed to AFt and unreacted gypsum. It has been shown that the transformation of calcium sulfite and calcium sulfate results in absorption peaks associated with the radical ion SO_2_^−^ near 460 cm^−1^ [34]. There is a weak peak near 462.83 cm^−1^, and the frequency increases to 464.76 cm^−1^ at 28 days.

#### 2.3.4. TG-DSC

**Figure 10:**
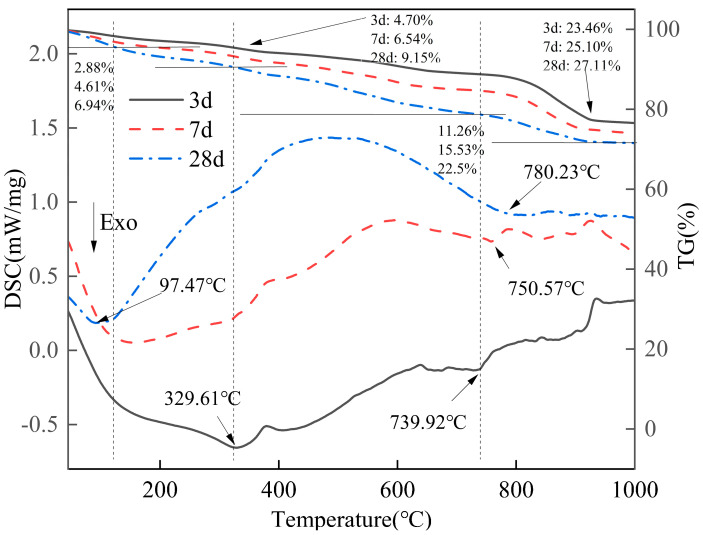
The TG-DSC curves of pastes at different curing age.

The TG-DSC curves of DA-BFS gelling materials are shown in Figure 10. There are three significant exothermic peaks in the DSC curves at different hydration ages. The first exothermic peak was located around 60~200 °C, and the mass loss rate was 2.88% at 3 d and 4.61% at 7 d. A significant exothermic peak appeared at 97.47 °C at 28 d with the generation of a large amount of C-S-H gel, at which time the mass loss rate was 6.94%. The second exothermic peak was located between 290 °C and 350 °C, and there was an obvious exothermic peak at 329.61 °C at 3 d with a mass loss rate of 4.70%. The exothermic peak at this stage was mainly caused by the dehydration reaction of AFt and C-S-H gel. The third exothermic peak appeared between 670 and 800 °C, which was mainly due to the decomposition of CaCO_3_ to produce CaO and CO_2_ gases, resulting in the reduction in material mass. At 3 days of hydration, the exothermic peak appeared at 739.92 °C, with a mass loss rate of 11.26%; at 7 days of hydration, the exothermic peak was located at 750.57 °C, with a mass loss rate of 15.53%; and at 28 days of hydration, the exothermic peak was at 780.23 °C, with a mass loss rate of 22.5%. This phenomenon shows that with hydration, calcium hydroxide undergoes carbonation reaction to generate calcium carbonate, and at the same time, due to the large number of calcium ions in the system, it is easy to absorb carbon dioxide to form calcium carbonate.

### 2.4. Reaction Mechanism

**Figure 11:**
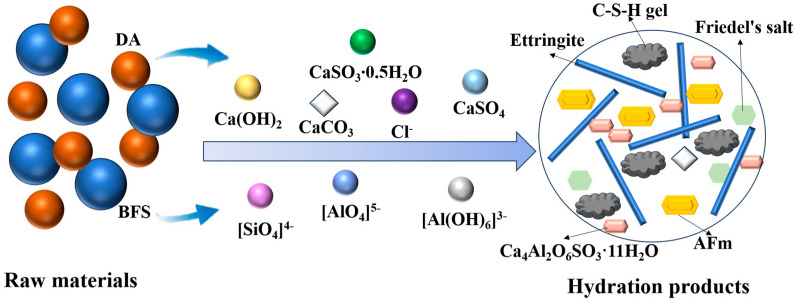
Hydration process of the BFS-DA gelling materials.

The hydration mechanism of BFS-DA gelling materials is shown in Figure 11. DA can perform alkali and sulfate activation for BFS. When BFS-DA gelling materials are mixed with water, the dissolution of Ca(OH)_2_ in DA has a high alkalinity, which promotes the depolymerization of silica–alumina in BFS as shown in Equations (1) and (2) [35]. The Si-O-Al bond breakage was confirmed in the FTIR analysis.

(1) $$2CaO\cdot SiO_{2}+H_{2}O\to 3Ca^{2+}+SiO_{4}^{2-}+2OH^{-}$$

(2) $$AlO_{2}^{-}+2OH^{-}+H_{2}O\to {\left[Al{\left(OH\right)}_{6}\right]}^{3-}$$

The SO_4_^2−^, Ca^2+^, and Cl^−^ formed by DA dissolution, and Al(OH)_6_^3−^, reacted to form ettringite and Friedel’s salt, as shown in Equation (3) [36] and Equation (4) [37].

(3) $$6Ca^{2+}+2{\left[Al{\left(OH\right)}_{6}\right]}^{3-}+3SO_{4}^{2-}+26H_{2}O\to Ca_{6}Al_{2}{\left(SO_{4}\right)}_{3}{\left(OH\right)}_{12}\cdot 26H_{2}O$$

(4) $$4Ca^{2+}+2Cl^{-}+2{\left[Al{\left(OH\right)}_{6}\right]}^{3-}+4H_{2}O\to 3CaO\cdot Al_{2}O_{3}\cdot 3CaCl_{2}\cdot 10H_{2}O$$

According to the XRD patterns and SEM images, the formation of ettringite in hydration is an important source of early strength. The formation of ettringite accelerates the depolymerization of silica–oxygen tetrahedra and aluminum–oxygen tetrahedra, which form C-S-H gel in alkaline environments as in Equation (5).

(5) $$Ca^{2+}+H_{3}SiO_{4}^{-}+H_{2}O\to C-S-H$$

As the reaction processed, the supply of SO_4_^2−^ was not sufficient to continue the formation of ethyl feldspar, and instead, AFm was formed as shown in Equation (6) [38]. In addition, CaSO_3_∙0.5H_2_O in DA also participates in the reaction to form Ca_4_Al_2_O_6_SO_3_·11H_2_O, as shown in Equation (7).

(6) $$4Ca^{2+}+2{\left[Al{\left(OH\right)}_{6}\right]}^{3-}+SO_{4}^{2-}+12H_{2}O\to Ca_{4}Al_{2}O_{6}SO_{4}\cdot 12H_{2}O$$

(7) $$4Ca^{2+}+2{\left[Al{\left(OH\right)}_{6}\right]}^{3-}+SO_{3}^{2-}+11H_{2}O\to Ca_{4}Al_{2}O_{6}SO_{3}\cdot 11H_{2}O$$

Extensive studies have reported the conversion of DA to calcium sulfite to calcium sulfate by thermal treatment at 170–500 °C for resource utilization [34,39]. It has also been shown that the hydration heat under composite synergistic hydration conditions can provide the conversion of DA from hannebachite to gypsum at low temperatures [18]. Although the chemical bonding of SO_2_^−^ characteristic of the conversion process was observed in the FTIR pattern of BFS-DA paste, no enhancement of the diffraction peaks of gypsum was found in the XRD pattern. This suggests that gypsum transformed by hannebachite is involved in the reaction to form a sulfate-containing mineral phase.

### 2.5. Economic and Environmental Benefits

Compared with cement, BFS-DA gelling materials have the advantages of economic and environmental benefits in raw material acquisition and production. BFS is widely used in cement mixing materials, and its price is about 50% of that of cement [40]. DA is the solid waste that iron and steel enterprises urgently need to deal with, so they are usually free or even have disposal subsidies of CNY 90 per ton. BFS-DA gelling materials satisfy the index requirements of GB/T 28294-2024, and the raw material has a lower cost than cement, so it has a broad application prospects and economic benefits. Cement produces about 500–700 kg/t of CO_2_ in the production process, while the production process of BFS-DA cementitious materials does not have high-temperature calcination, so it is a kind of environmentally friendly and low-carbon building material.

## 3. Conclusions

In this study, the feasibility of BFS synergized with DA for the preparation of gelling materials was evaluated and the hydration mechanism of BFS-DA gelling materials was illustrated. This provides a new direction for the large-scale comprehensive utilization of DA, and the main conclusions are as follows:

(1) Experiments on the proportion of raw materials were carried out to determine the optimum proportion of BFS and DA, which were 60% and 40%, respectively, in the absence of any stimulant. The compressive strengths of 3 d, 7 d, 28 d, and 56 d samples were 14.21 MPa, 20.24 MPa, 43.50 MPa, and 46.27 MPa, respectively, and the initial and final setting times were 495 min and 570 min, respectively, and the stability was qualified to meet the requirements of GB/T 28294-2024.

(2) Mixed grinding of BFS and DA significantly enhanced the activity of BFS-DA gelling materials compared to grinding DA individually. The increase in compressive strength was 89.86%, 66.36%, 24.56%, and 25.68% at 3, 7, 28, and 90 d after 30 min of mixed grinding, and the compressive strengths reached 26.22 MPa, 35.6 MPa, 58.33 MPa, and 63.97 MPa, respectively.

(3) Mixed grinding can shorten the hydration induction period of BFS-DA slurry and enhance the exothermic rate. The cumulative heat of hydration of BFS-DA slurry is about 120 J/g, which is 61.3% lower than that of OPC, and it can be used to prepare concrete with relatively low heat of hydration.

(4) The main hydration products of BFS-DA gelling materials include ettringite, C-S-H gel, AFm, and Friedel’s salt. Calcium sulfite in DA participates in the hydration to form Ca_4_Al_2_O_6_SO_3_·11H_2_O, providing strong evidence for the direct use of DA in the preparation of gelling materials.

## 4. Materials and Methods

### 4.1. Raw Materials

**Figure 12:**
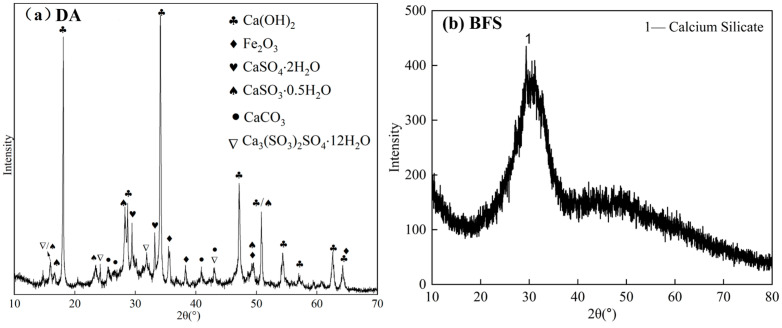
XRD patterns of (**a**) DA, and (**b**) BFS.

Semi-dry flue gas desulphurization ash (DA, Anshan Iron and Steel Pelletizing Plant) and granulated blast furnace slag (BFS, Anshan Iron and Steel Green Source Technology Co., Ltd.) comprise the raw materials for the gelling materials. The chemical compositions of the raw materials are shown in Table 4, and the mineral compositions are shown in Figure 12. The DA from steel plant production has a calcium-based compound as its main crystalline phase. The main chemical compositions of DA are CaO and SO_3_, accounting for 47.61% and 24.76%, respectively. The main mineral compositions of DA are Ca(OH)_2_, hannebachite, and calcite. The main chemical compositions of BFS are CaO, SiO_2_, Al_2_O_3_, and MgO, accounting for 48.70%, 30.26%, 9.97%, and 5.7%, respectively. According to the alkalinity coefficient Equation (8), the *Mo* of BFS was calculated to be 1.35, which is an alkaline slag. The *K* (Equation (9)) of BFS was calculated to be 2.08 according to the quality coefficient formula, which belongs to high-activity slag.

(8) $$M_{o}=\frac{w_{\mathrm{Cao}}{+w}_{\mathrm{MgO}}}{w_{{\mathrm{SiO}}_{2}}{+w}_{{\mathrm{Al}}_{2}O_{3}}}$$

(9) $$K=\frac{w_{\mathrm{Cao}}{+w}_{\mathrm{MgO}}{+w}_{{\mathrm{Al}}_{2}O_{3}}}{w_{{\mathrm{SiO}}_{2}}{+w}_{\mathrm{MnO}}{+w}_{{\mathrm{TiO}}_{2}}}$$

**Figure 13:**
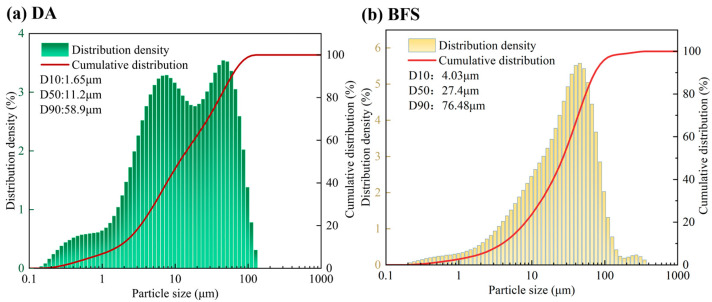
Particle size distribution of DA and BFS.

Particle size analyses of the DA and BFS samples are shown in Figure 13. The particle size distribution of DA shows a broad bimodal pattern, with a wide range of fine particles from 1.6 μm to around 50 μm, and with 90% of the total number of particles in the range of 1~60 μm. BFS has a single-peaked size distribution, with the smallest size of 4.03 μm, the largest of 76.48 μm, and an average particle size of 27.4 μm. The density of DA is 2.68 g/cm^3^, the specific surface area is 383 m^2^/kg, and the pH value is 12.44. In addition, the measured density of BFS was 2.81 g/cm^3^ and the specific surface area was 400 m^2^/kg.

### 4.2. Methods

#### 4.2.1. Preparation of Gelling Materials with Different DA Dosages

All-solid waste gelling materials were prepared by mixing BFS and DA according to the dosage and water–solid ratio in Table 5. The pastes specimens were poured into 30 mm × 30 mm × 50 mm molds and placed in a curing chamber with a relative humidity of 95% ± 2% and a temperature of 25 ± 2 °C. After removing the molds, the pastes specimens will be tested for strength at different curing times (3, 7, 28, and 56 days).

#### 4.2.2. Different Mechanical Grinding Experiments

Two types of grinding were used in this study: (1) DA was individually ground within a laboratory small mill (SMΦ500 × 500, Daohui Town Fusheng Assay Instrument Factory, Shaoxing, China) and activated for 15 min, 25 min, 30 min, 40 min, and 50 min; (2) DA and BFS were mixed according to the optimal mixing ratio and then mixed and ground for 30 min, 40 min, and 50 min, respectively. The mechanically milled material was sealed and preserved. At the end of each grinding, a meticulous evaluation was performed to scrutinize the size and specification of the particles. A laser particle size distribution meter (BT-9300SE, Suzhou, China) and a specialized specific surface area meter (CZB-9, Tianjin, China) were used. The preparation of paste specimens is as mentioned in Section 4.2.1.

#### 4.2.3. Strength Testing, Flowability, Setting Time, Stability, and Hydration Heat

The compressive strength test was conducted using three test blocks per age, measured with a YES-300 digital hydraulic testing machine (Changchun No.1 Material Testing Machine Factory).

The flowability of pastes refers to GB/T 8077 [41]. The well-mixed DA-BFS slurry was divided into two layers and placed into the mold of the flow test apparatus. The top opening of the mold is 70 mm in diameter, the bottom opening is 100 mm in diameter, and the height is 60 mm. The slurry was compacted in the mold, and 25 jumps were completed within 25 ± 1 s. At the end of the experiment, the diameter of the bottom of the mortar was measured with a caliper and repeated three times for each group.

To assess the setting time of BFS-DA gelling materials, according to GB/T 1346 standard [42], the setting time of fresh slurry was measured using a Vicat instrument (DL-AWK, Beijing, China). Before the test, install the test needle and set the device to zero position. Fresh paste was injected into a conical mold and the surface of the mold was gently vibrated and scraped. Determine the set state of the paste by observing the scale reading. When the scale reading is in the range of 3 to 5 mm, the paste has reached the initial solidification state. In the same procedure, the slurry is considered to have reached final set when the scale of the final set test needle reads 0.5 mm.

According to the test method of cement autoclave expansion in GB/T 750 standard [43], the expansion rate of BFS-DA gelling materials was tested. The boiling stability of BFS-DA gelling materials was tested according to GB/T 1346 standard.

Hydration heat of pastes was measured using an isothermal calorimeter (IC, TAM Air 8) at 20 degrees Celsius. To illustrate the effect of mixing and grinding on the activation of gelling materials, mixtures of various gelling materials were prepared by mixing and grinding for 30, 40, and 50 min. Water was added to the homogenized gelling mixture, and pastes were stirred externally for 2 min. The homogenate (approximately 1 g) was then poured into an ampoule, sealed, and placed in the test chamber. During a period of 144 h, the heat flux of each paste was recorded.

#### 4.2.4. Microanalysis

The hydration products of the pastes were identified by X-ray diffraction (XRD). X-ray diffraction analysis was performed on an Ultima IV X-ray diffractometer (Akishima, Tokyo, Japan). It was carried out at 30 mA and 50 kV. The step interval of 2θ is 0.02° from 5° to 70°. A synchronized thermal analysis (Thermogravimetric Analysis-Differential Scanning Calorimetry, TG-DSC) was carried out on a NETZSCHSTA 409-QMS (Setaram Instruments Ltd., Lyon, France) to measure the heat change and mass loss of the hydrated pastes at different temperatures in the presence of inert nitrogen (N_2_). The test temperature was increased from 20 °C to 1000 °C at a rate of 5 °C per minute. An FTIR analysis of the hydrated products was performed with a NEXUS-670 spectrometer (ThermoFisher, Maltham, MA, USA), operating at a resolution of 3 cm^−1^ and a wavenumber range of 400–4000 cm^−1^. A field emission scanning electron microscope (FE-SEM, type SUPRA55, Carl Zeiss, Oberkochen, Germany) was used to visualize the microstructure of the hydrated pastes on the fracture surface of the carbon coating. The operating voltage was 10 kV and the vacuum was below 9.9 × 10^−6^ mbar. An energy dispersive X-ray spectrometer (EDX, Carl Zeiss, Oberkochen, Germany) was equipped on the FE-SEM to analyze the chemical composition of the hydration products.

## Figures and Tables

**Table 1:** Characteristic particle sizes of BFS-DA gelling materials with different degrees of fineness.

Grinding Time (min)	Specific Surface Area (m^2^/kg)	Particle Size Parameters (μm)		
		D10	D50	D90
0	395	2.34	17.19	74.60
30	580	0.65	9.64	68.00
40	591	0.62	8.55	58.30
50	578	0.63	8.56	59.30

**Table 2:** Setting time and stability of BFS-DA gelling materials.

Sample	Initial Setting Time (min)	GB/T 28294	Final Setting Time (min)	GB/T 28294	Autoclave Expansion (%)	GB/T 28294	Soundness
M0	495	≥45	570	≤600	0.35	≤0.50%	compliant with the standard
M-40	451	≥45	563	≤600	0.25	≤0.50%	compliant with the standard

**Table 3:** EDX element composition of the compounds in SEM images (wt.%).

	Compounds	Ca	O	Al	Si	S	Fe
Point 1	Portlandite	45.07	54.93		-	-	-
Point 2	Ettringite	63.81	7.71	5.88	14.05	6.25	0.47
Point 3	C-S-H gel	18.78	43.35	3.76	33.33	0.58	0.60
Point 4	AFm	26.97	56.30	10.44	-	5.67	-

**Table 4:** Chemical composition (wt%) of DA and BFS.

Sample	CaO	SiO_2_	Al_2_O_3_	MgO	SO_3_	TiO_2_	Na_2_O	MnO	Fe_2_O_3_	K_2_O	Cl	LOI
DA	47.61	5.5	0.52	1.06	24.76	-	1.33	1.06	8.16	6.36	1.58	2.05
BFS	48.70	30.26	9.97	5.70	2.39	0.39	0.26	0.18	0.49	0.42	0.03	1.27

**Table 5:** Mixture proportions of designed gelling materials (wt%).

	DA	BFS	W/S
G1	25	75	0.32
G2	30	70	0.32
G3	35	65	0.32
G4	40	60	0.32
G5	45	55	0.32
G6	50	50	0.32
G7	55	45	0.32
G8	60	40	0.32

## Data Availability

The original contributions presented in this study are included in the article. Further inquiries can be directed to the corresponding authors.
